# Analysis of risk factors for recurrence of Budd–Chiari syndrome: A retrospective study with zero-inflated model

**DOI:** 10.1097/MD.0000000000045511

**Published:** 2025-11-21

**Authors:** Shengli Li, Xiangting Liu, Muyao Zhou, Cuocuo Wang, Hui Wang, Qingqiao Zhang, Lei Wang, Yong Zhou

**Affiliations:** a Clinical Research Institute, The Affiliated Hospital of Xuzhou Medical University, Xuzhou, Jiangsu, China; b School of Computer Science and Technology/School of Artificial Intelligence, China University of Mining and Technology, Xuzhou, Jiangsu, China; c Department of Geriatrics, Xuancheng People’s Hospital, Xuancheng, Anhui, China; d Jiangsu Normal University, Xuzhou, Jiangsu, China; e Department of Hepatobiliary Surgery, Xuzhou Central Hospital, Xuzhou, Jiangsu, China; f Department of Interventional Radiology, The Affiliated Hospital of Xuzhou Medical University, Xuzhou, Jiangsu, China.

**Keywords:** Budd–Chiari syndrome, dispersion, recurrence, ZINB regression

## Abstract

This study aims to identify an optimal model for assessing the recurrence frequency of Budd–Chiari syndrome (BCS) and further analyze the factors contributing to BCS recurrence. A total of 754 patients who were admitted to the Affiliated Hospital of Xuzhou Medical University between January 2015 and July 2022 were included. We constructed 4 different count outcome models: Poisson, negative binomial (NB) model, zero-inflated Poisson (ZIP) model, and zero-inflated negative binomial (ZINB) model. We selected the model with the best fitting performance to explore factors associated with BCS recurrence. Of 754 respondents, 511 reported no recurrence. Log-likelihood ratio tests indicated that the NB model performed better than the Poisson regression model (χ^2^ = 124.91, *P* < .001), and the ZINB model outperformed the ZIP model (χ^2^ = 34.29, *P* < .001). In the ZINB model, the analysis of the counting process revealed that the variables significantly associated with recurrence frequency included age (odds ratio [OR] = 0.69; 95% confidence interval [CI]: 0.57–0.84), sex (female: OR = 1.77; 95% CI: 1.24–2.55), anticoagulant use (warfarin vs new oral anticoagulants [NOACs]: OR = 2.11, 95% CI: 1.34–3.31; not using anticoagulants vs NOACs: OR = 1.98, 95% CI: 1.20–3.28), absence of cirrhosis (OR = 0.57, 95% CI: 0.40–0.82), and neutrophil count (NEU) (OR = 1.22, 95% CI: 1.04–1.42). The zero-inflated model proved robust in identifying factors influencing BCS recurrence compared with the other models. Influence of sex, surgery, anticoagulation, cirrhosis, hospital duration, apolipoprotein A, and NEU on the recurrence risk and frequency of BCS.

## 1. Introduction

Budd–Chiari syndrome (BCS) is a rare yet fatal vascular disorder characterized by hepatic venous outflow tract obstruction. This obstruction is independent of its level or underlying mechanism, provided that it is not attributed to pericardial disease, cardiac conditions, or veno-occlusive disease.^[1]^ China has the highest number of reported BCS cases worldwide. With an estimated annual incidence of 0.88 per million and prevalence of 7.69 per million, China hosts a significant population of over 20,000 documented patients with BCS, primarily concentrated in the Huanghuai region.^[2]^ In contrast to Europe and India, where over 80% of BCS cases are associated with hypercoagulable states, the pathogenesis of BCS in China predominantly follows a dynamic pathological process characterized by “vascular endothelial damage – inflammatory reaction – endothelial recovery and intimal hyperplasia – thrombus formation and organization – membrane formation.”^[3]^ The European Association for the Study of the Liver recommends a step-wise therapeutic approach for BCS treatment,^[4–6]^ which includes: medical therapy, percutaneous recanalization of hepatic veins (HVs) and/or the inferior vena cava (IVC), placement of a transjugular intrahepatic portosystemic shunt (TIPS), and orthotopic liver transplantation. Thrombolytic therapy is recommended in case of acute BCS with an identifiable clot.

However, a multi-center European study involving 157 patients with BCS managed through this step-by-step strategy reported discouraging the 5-year intervention-free survival rates.^[7]^ In India, the 5-year cumulative patency rates for recanalization and TIPS were 74% and 68%, respectively, among 510 patients receiving endovascular interventions.^[8]^ In China, the reported rates of restenosis or occlusion range from 20 to 40%,^[9–11]^ posing a significant threat to public health. In recent years, the incidence of BCS has been steadily increasing in Shan dong and the northern region of Jiangsu.^[2,12,13]^ With an anticipated increase in life expectancy, the number of recurrent cases is expected to increase, thereby worsening the societal and medical burdens. Therefore, it is imperative to identify the underlying risk factors of BCS recurrence to enhance surveillance and facilitate early intervention. Traditional statistical methods, such as logistic regression and Cox proportional hazards models, have been employed to evaluate the prognosis. Recently, a nomogram model based on Cox regression was established to predict the first recurrence of BCS after endovascular treatment.^[14]^ However, conventional regression models are unsuitable for handling data that have a substantial proportion of zeroes.

To address these limitations, alternative modeling approaches, such aszero-inflated or hurdle models, are frequently used. Zero-inflated explicitly accounts for excess zeros by incorporating 2 components: the first group generates an all-zero subset, whereas the second group follows a Poisson or negative binomial (NB) distribution for “non-zero” outcomes.^[15]^ These extended models have found widespread application in areas such as microbiome research, infectious diseases, epidemics, and chronic diseases.^[16,17]^ Therefore, we aimed to identify relevant risk factors associated with BCS recurrence based on the best-fitting model. This analysis provides a valuable reference for formulating early personalized intervention plans for patients with BCS, marking a significant step toward the realization of precision medicine.

## 2. Methods

### 2.1. Patients

This retrospective investigation included 754 patients with BCS admitted to the Affiliated Hospital of Xuzhou Medical University between January 2015 and July 2022. BCS was diagnosed using color Doppler ultrasonography, multimodal computed tomography, magnetic resonance imaging, and/or angiography.^[18]^ Exclusion criteria were as follows: secondary BCS; hepatic outflow obstruction attributed to congestive heart disease, sinusoidal obstruction syndrome, or other causes; substantial dysfunction of vital organs such as the liver, kidney, and brain, or malignancies, including liver cancer; non-standardized anticoagulation; and Patients lost to follow-up or who died prior to recurrence. The study protocol was approved by the Ethics Committee of the Affiliated Hospital of Xuzhou Medical University (Jiangsu, China; Approval No. XYFY2023-KL188-01). Given the retrospective design and the use of anonymized clinical data, the committee waived the requirement for informed consent.

### 2.2. Data collection

The collected data included recurrence frequency, age, sex, occupation (farmers or workers), BCS classification, type of operation, anticoagulant use, presence of liver cirrhosis, thrombosis status, hospital duration, neutrophil count (NEU), platelet count, prothrombin time (PT), activated partial thromboplastin time (APTT), fibrinogen, thrombin time, aspartate aminotransferase, alanine aminotransferase, albumin (ALB), glucose (GLU), total bilirubin (TBIL), cholesterol, triglyceride, apolipoprotein A (ApoA), apolipoprotein B, lipoprotein(a), high-density lipoprotein (HDL), low-density lipoprotein, cystatin C, lactate dehydrogenase (LDH) and alpha-fetoprotein. BCS is classified into 3 main types^[19]^: HV, IVC, and mixed (HV + IVC). Operations were categorized into 5 main forms: simple balloon dilation, stent implantation, catheter-directed thrombolysis, TIPS, and conservative treatment. The anticoagulant options included warfarin, new oral anticoagulants (NOACs), and no anticoagulants.

### 2.3. Follow-up

Recurrence was defined as the presence of a newly thrombosed vessel or partial/complete occlusion in the IVC, HV, or collateral veins, with reversed or nonexistent flow. Additionally, new symptoms such as recurrent ascites, abdominal wall varicosis, lower limb swelling or upper gastrointestinal bleeding were considered indicative of recurrence after the patient’s condition had initially stabilized.^[14,20]^ Patients were subjected to regular 6-month follow-ups from July 2021 until the completion of the study in July 2023. Telephone communication and outpatient records were used to establish contact with patients or their family members. Respondents were divided into 2 groups: those with recurrence and those without recurrence. During each 6-month follow-up visit, patients underwent clinical assessment and routine Doppler ultrasound to evaluate hepatic venous and IVC flow.

### 2.4. Related basic principles

#### 2.4.1. Overdispersion test

The *O*-test is based on mean and variance testing to determine whether there is over dispersion. For excessively discrete count data, Poisson regression often underestimates the standard error (SE) of parameter estimates, leading to larger statistical quantities, thereby increasing Type I errors and exaggerating the effects of the explanatory variables. The calculation formula is as follows:


$$\begin{array}{l} O=\sqrt{\frac{n-1}{2}}\left(s^{2}-\bar{x}\right)/\bar{x} \end{array}$$

where n represents the total number of patients, and and are the variance and mean of the number of relapses, respectively. When the absolute value of the *O* statistic is >1.96 and the mean is much smaller than the variance, it indicates that the data has over discreteness. Continuing to use the Poisson distribution will result in a decrease in the SE and estimation efficiency, whereas using the NB distribution is more reasonable. When <1.96, it cannot be considered that the variance is significantly greater than the mean, and there is no dispersion in the data.

#### 2.4.2. Vuong test

The Vuong test was suitable for comparing the goodness-of-fit of the 2 non-nested models. The calculation formula is as follows:


$$\begin{array}{l} V=\frac{\sqrt{n}\left[\left(1/n\right)\overset{n}{\underset{i=1}{\sum }}m_{i}\right]}{\sqrt{\left(1/n\right)}\overset{n}{\underset{i=1}{\sum }}{\left(m_{i}-\bar{m}\right)}^{2}}=\frac{\sqrt{Nm}}{S_{m}} \end{array}$$

When *V* < −1.96, it is recommended to choose Poisson or NB; when *V* > 1.96, it indicates zero inflation in the data, and it is recommended to choose a zero inflation model; when | *V* |<1.96, it is impossible to determine which model is better and other means are needed. The Vuong package in R was used for the calculations.

#### 2.4.3. LR test

Assuming that Model 1 is nested within Model 2, the LR test can be used for the model selection. The corresponding statistics are as follows:


$$\begin{array}{l} LR=-2\left(lnL_{2}-lnL_{1}\right) \end{array}$$

The LR follows a χ^2^ distribution with a degree of freedom of *v*, which is the number of restricted parameters. If the LR value is <0.05 under the chi-square distribution, it indicates that Model 1 is superior to Model 2. The lrtest() function of the lmtest package in R was used to complete it.

### 2.5. Statistical analyses

All statistical analyses were conducted using R version 4.0.3 (https://www.rproject.org/) with the following packages: mice, nnet, glmnet, glm, glmmTMB, MASS, pscl, lmtest, and ggplot2. Statistical significance was set at *P* < .05. The predictive mean matching method was employed to address missing data, with imputation performed across 5 imputation matrices, iterated over 50 cycles. Details on the extent of missing data were provided in Table S1 (Supplemental Digital Content, https://links.lww.com/MD/Q587). Categorical variables are presented as counts, whereas categorical variables are expressed as means and standard deviations. The *t*-test was used for continuous variables, and the χ^2^ test was used for categorical variables. Variance inflation factor analysis was used to assess collinearity between variables, with variables selected based on elastic net techniques.^[21,22]^ *Z*-score transformation was employed to standardize the laboratory indicator data and eliminate dimensional differences.

The entire dataset was randomly divided into training (n = 603) and validation (n = 151) sets at a 7:3 ratio. Over-dispersion was detected using the *O* statistic. Poisson, NB, and zero-inflated regression models with identical variables were fitted to the data. Vuong statistics were used to compare the goodness of fit between the zero-inflation model and corresponding traditional models. Similarly, the log-likelihood ratio (LR) test allows for comparisons of nested models (Poisson vs NB and zero-inflated Poisson (ZIP) vs zero-inflated negative binomial (ZINB)). The selection of the optimal model relied on the minimum values of the Akaike information criteria (AIC), corrected AIC (AICc), and −2LogLikelihood (−2LL). Additionally, the root mean squared error, mean absolute error, accuracy, precision, and predictive curve are employed to evaluate the forecasting performance.

## 3. Results

### 3.1. Patient characteristics

**Figure 1.**
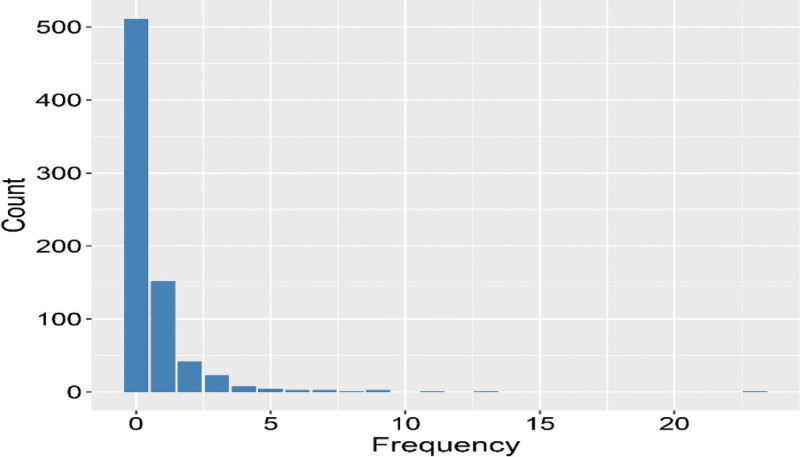
Recurrence frequency histogram.

Of the initial 1300 patients, 454 were excluded for various reasons, including malignancies (n = 202), liver and kidney failure (n = 85), hepatic encephalopathy (n = 25), secondary BCS (n = 30), and irregular anticoagulant therapy (n = 112). In addition, 92 patients were lost to follow-up before recurrence (Fig. S1, Supplemental Digital Content, https://links.lww.com/MD/Q587). In total, 754 patients were included in the modeling. A histogram (Fig. 1) depicting the recurrence frequency of these patients revealed that 511 patients reported no recurrence, 157 experienced recurrence once, 49 experienced 2 recurrences, and 28 experienced 3 recurrences during the follow-up period. The variance (2.46) exceeded the mean (0.64), and the *O* statistic was 55.08 with a *P*-value of <.001, indicating data over-dispersion. Notably, high-risk areas were primarily concentrated in the Huanghuai region, particularly in the Jiangsu (n = 295), Shandong (n = 140), Anhui (n = 131), and Henan (n = 55) provinces. The top 3 recurrent symptoms were abdominal distension and pain, lower-limb swelling, and abdominal wall varicose veins.

Table 1 shows the demographic and clinical characteristics of the patients. The male-to-female ratio was 1.32:1, with a mean age of 48.80 ± 13.22 years (range: 12–82 years). Farmers and workers represented the majority, accounting for 46.15% of patients. Percutaneous angioplasty with or without stent placement is the predominant therapeutic modality for BCS in Turkey. In particular, 502 patients underwent balloon dilation, 78 stent implantation, 45 underwent thrombolytic therapy, 27 underwent stent placement, and 102 underwent conservative treatment. A total of 555 patients regularly used oral anticoagulants, including warfarin, rivaroxaban, apixaban and dabigatran. More than half of the patients had comorbid liver cirrhosis, and 141 patients presented with thrombosis. Significant differences in age, occupation, BCS type, operation type, anticoagulant use, and hospitalization duration. NEU, platelet count, PT, APTT, ALB, TBIL and HDL levels were compared between the relapse and non-relapse groups.

**Table 1:** Baseline characteristics in the study cohort.

Variables	Total	Not-recurrent	Recurrent	*P* value
	n = 754	n = 511	n = 243	
Age (yr)	48.80 ± 13.22	50.91 ± 12.36	44.34 ± 13.89	<.001
Sex (male/female)	429/325	278/233	151/92	.054
Laborer (yes/no)	348/406	260/251	88/155	<.001
Type (HV/IVC/MIX)	463/173/118	342/101/68	121/72/50	<.001
Cirrhosis (yes/no)	441/313	309/202	132/111	.128
Thrombus (yes/no)	141/613	86/425	55/188	.070
Operation (PTA/stent/thrombolysis/TIPS/none)	502/78/45/27/102	337/61/24/14/75	165/17/21/13/27	.011
Anticoagulants (warfarin/NOACs/none)	389/166/199	266/99/146	123/67/53	.019
Hospital duration (d)	11.22 ± 6.25	10.71 ± 5.52	12.30 ± 7.47	.001
NEU (10^9^/L)	2.81 ± 1.88	2.69 ± 1.81	3.06 ± 2.00	.013
PLT (10^9^/L)	120.59 ± 68.69	117.18 ± 63.13	127.76 ± 78.77	.048
PT (s)	14.47 ± 5.46	14.13 ± 5.36	15.20 ± 5.59	.012
APTT (s)	34.26 ± 9.61	33.55 ± 8.73	35.75 ± 11.12	.003
FIB (g/L)	2.37 ± 0.81	2.34 ± 0.74	2.44 ± 0.92	.104
TT (s)	17.60 ± 5.18	17.38 ± 1.58	18.07 ± 8.83	.085
AST (U/L)	33.29 ± 60.34	32.85 ± 70.59	34.20 ± 28.76	.774
ALT (U/L)	25.99 ± 33.52	25.03 ± 37.10	28.00 ± 24.26	.256
ALB (g/L)	40.75 ± 6.40	41.22 ± 6.30	39.77 ± 6.53	.004
TBIL (µmol/L)	33.16 ± 35.96	31.17 ± 31.19	37.34 ± 44.14	.028
CysC (mg/L)	0.92 ± 0.30	0.93 ± 0.33	0.90 ± 0.22	.370
GLU (mmol/L)	5.22 ± 1.82	5.21 ± 1.67	5.24 ± 2.10	.871
CHOL (mmol/L)	3.53 ± 0.93	3.57 ± 0.94	3.44 ± 0.93	.072
TG (mmol/L)	0.85 ± 0.41	0.85 ± 0.40	0.86 ± 0.42	.684
ApoA (g/L)	1.03 ± 0.29	1.04 ± 0.28	1.00 ± 0.31	.101
ApoB (g/L)	0.64 ± 0.21	0.64 ± 0.22	0.64 ± 0.21	.727
LPa (mg/L)	168.54 ± 152.71	162.16 ± 141.79	181.95 ± 173.01	.096
HDL (mmol/L)	1.20 ± 0.43	1.22 ± 0.43	1.14 ± 0.43	.01
LDL (mmol/L)	1.95 ± 0.76	1.96 ± 0.80	1.91 ± 0.66	.390
LDH (U/L)	212.47 ± 105.15	208.72 ± 99.22	220.34 ± 116.47	.156
AFP (ng/mL)	5.85 ± 28.86	5.26 ± 17.18	7.10 ± 44.34	.413

AFP = alpha-fetoprotein, ALB = albumin, ALT = alanine aminotransferase, ApoA = apolipoprotein A, ApoB = apolipoprotein B, APTT = activated partial thromboplastin time, AST = aspartate aminotransferase, CHOL = cholesterol, CysC = cystatin C, FIB = fibrinogen, GLU = glucose, HDL = high-density lipoprotein, HV = hepatic vein, IVC = inferior vena cava, LDH = lactate dehydrogenase, LDL = low-density lipoprotein, LPa = lipoprotein(a), NEU = neutrophil count, NOACs = new oral anticoagulants, PLT = platelet count, PT = prothrombin time, TBIL = total bilirubin, TG = triglyceride, TIPS = transjugular intrahepatic portosystemic shunt, TT = thrombin time.

### 3.2. Screen for relapse-related risk factors

**Figure 2.**
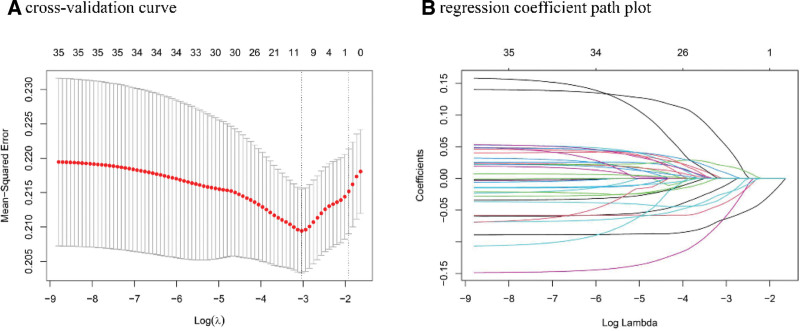
Lasso regression cross-validation method for analyzing clinical features. (A) Cross-validation curve illustrating the mean squared error as a function of the logarithm of lambda. (B) Regression coefficient path plot showing how the coefficients of the variables change with the log(λ) values.

The variance inflation factors corresponding to operation type, anticoagulant use, APTT, aspartate aminotransferase, alanine aminotransferase, cholesterol, triglyceride, HDL, low-density lipoprotein, ApoA, and apolipoprotein B levels exceeded 2, indicating multicollinearity. The elastic net method was employed to compress the regression coefficients of unnecessary variables to zero and subsequently eliminate them from the model for variable screening. Figure 2 shows the selection of potential predictors through the least absolute shrinkage and selection operator regression. 9 variables with non-zero coefficients were selected based on the minimum lambda, including age, sex, occupation, operation type, anticoagulant use, NEU, ALB, GLU, and LDH. In addition, the influence of liver cirrhosis, BCS type, and elevated TBIL levels on patient prognosis has been confirmed.^[9,14,23–27]^ Furthermore, Talen suggested that decreased ApoA levels may play a role in the etiology of thrombosis in patients with BCS and potentially in other patients with venous thrombosis.^[28]^ Thus, the variables of age, sex, occupation, operation type, anticoagulant use, NEU, ALB, GLU, LDH, liver cirrhosis, BCS type, and TBIL were used to build models in the training set.

### 3.3. Modeling and comparison

**Figure 3.**
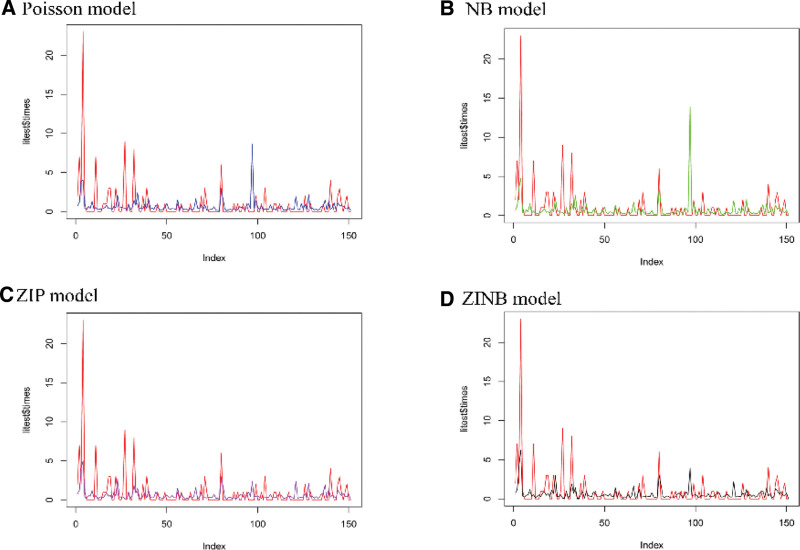
Comparison of residual plots for 4 count outcome models: (A) Poisson model, (B) negative binomial (NB) model, (C) zero-inflated Poisson (ZIP) model, and (D) zero-inflated negative binomial (ZINB) model. The x-axis represents the patient index (sorted by observation order), and the y-axis shows the deviance residuals (unitless). Different colored lines represent residuals from multiple imputation iterations, indicating variation across imputed datasets.

Non-nested models (i.e. Poisson vs ZIP and NB vs ZINB) were compared using the Vuong test. The results of the Vuong test provide evidence for the preference of ZIP over Poisson (*z* = 4.11, *P* < .001) and ZINB over NB (*z* = 3.40, *P* < .001) (Table 2). For nested models, the log-likelihood of the Poisson model (LL = −635.40) was greater than that of NB (LL= −572.94) with a statistic of 124.91 (*P* < .001), which revealed that the latter was better. Similarly, ZIP (LL = −567.27) was inferior to ZINB (LL = −550.13) with a statistic of 34.29 (*P* < .001). ZINB exhibited the smallest −2LL (1100.26), AIC (1182.26), AICc (1188.40), mean absolute error (0.94), and root mean squared error (2.02), and the highest accuracy (58.94%) and precision (28.07%) (Table 3). As shown in Figure 3, the prediction effect of the zero-inflated model is consistent with the actual values compared to the traditional count models. Finally, the ZINB model was chosen as the optimal model for the further analysis of recurrence-related indications.

**Table 2:** Vuong test and LR test results.

	Relationship	Vuong test		Likelihood ratio test	
		*z*	*P*-value	χ^2^	*P*-value
NB vs Possion	Test	–	–	124.91	<.001
ZINB vs ZIP	Test	–	–	34.29	<.001
ZIP vs Possion	Non-test	4.11	<.001	–	–
ZINB vs NB	Non-test	3.40	<.001	–	–

LR = log-likelihood ratio, NB = negative binomial, ZINB = zero-inflated negative binomial, ZIP = zero-inflated Poisson.

**Table 3:** Comparison of the fitness indicators and predictive indicators for 4 models.

	Fitness indicators				Predictive indicators			
	AIC	AICc	BIC	−2LL	RMSE	MAE	Accuracy	Precision
Possion	1310.80	1312.24	1398.84	1270.80	2.23	1.01	56.29%	23.73%
NB	1187.89	1189.48	1280.33	1145.89	2.36	1.02	56.29%	24.14%
ZIP	1214.55	1220.38	1390.62	1134.55	2.08	0.96	55.63%	23.21%
ZINB	1182.26	1188.40	1362.74	1100.26	2.02	0.94	58.94%	28.07%

AIC = Akaike information criterion, BIC = Bayesian information criterion, MAE = mean absolute error, NB = negative binomial, RMSE = root mean squared error, ZINB = zero-inflated negative binomial, ZIP = zero-inflated Poisson.

### 3.4. Result of the ZINB model

The coefficients, corresponding SEs, *z*-scores, and *P*-values for each variable in the ZINB model are listed in Table 4. In the zero section on the right side, sex, surgery, anticoagulant use, liver cirrhosis, hospital duration, and ApoA were determinants of recurrence. The number of BCS recurrences was more likely to be zero among women (odds ratio [OR] = 22.43, 95% confidence interval [CI]: 2.41–208.46). Use of warfarin (OR = 7.10, 95% CI: 1.12–45.15) or no anticoagulants use (OR = 14.51, 95% CI: 2.33–90.24) exhibited a higher risk of developing recurrence than NOACs. Furthermore, not having liver cirrhosis (OR = 0.15, 95% CI: 0.04–0.62), long hospital duration (OR = 0.4, 95% CI: 0.20–0.81), and higher levels of ApoA (OR = 0.37, 95% CI: 0.18–0.74) were associated with increased odds of reporting zero recurrence with BCS. Patients who underwent stent implantation had a significantly higher risk of recurrence than those who underwent simple balloon dilatation (OR = 17.49, 95% CI: 1.32–231.99). The results from the count section revealed that age, sex, anticoagulation, presence or absence of cirrhosis, and NEU exerted significant effects on the recurrence frequency of BCS. Older patients (OR = 0.69, 95% CI: 0.57–0.84) and those without liver cirrhosis (OR = 0.57, 95% CI: 0.40–0.82) showed fewer relapses. However, female patients (OR = 1.77, 95%CI: 1.24–2.55), patients using warfarin or no anticoagulants (OR = 2.11, 95% CI: 1.34–3.31), and patients with an increased NEU (OR = 1.98, 95% CI: 1.20–3.28) showed more relapse times.

**Table 4:** Zero-inflated negative binomial regression analysis.

Variables	Count component					Zero component				
	Estimate	SE	*z* value	*Pr* (>*z*)	OR (95% CI)	Estimate	SE	*z* value	*Pr* (>*z*)	OR (95% CI)
(Intercept)	−0.63	0.21	−3.09	<0.05	0.53 (0.35–0.79)	−3.21	1.36	−2.37	<0.05	0.04 (0.00–0.58)
Age	−0.37	0.1	−3.61	<0.001	0.69 (0.57–0.84)	0.3	0.34	0.87	0.38	–
Sex										
Female (vs male)	0.57	0.19	3.1	<0.05	1.77 (1.24–2.55)	3.11	1.14	2.74	<0.01	22.43 (2.41–208.46)
Laborer										
Yes (vs No)	−0.26	0.18	−1.45	0.15	-	−0.66	0.64	−1.03	0.3	–
Type										
HV (vs IVC)	0.18	0.22	0.8	0.43	–	−0.95	0.73	−1.31	0.19	–
MIX (vs IVC)	−0.11	0.25	−0.45	0.65	–	−0.77	0.9	−0.86	0.39	–
Operation										
Stent (vs PTA)	0	0.41	0.01	0.99	–	2.86	1.32	2.17	<0.05	17.49 (1.32-231.99)
Thrombolysis (vs PTA)	0.3	0.28	1.07	0.29	–	−17.9	3849.21	−0.01	1	–
TIPS (vs PTA)	−0.74	0.4	−1.82	0.07	–	−2.85	1.85	−1.54	0.12	–
None (vs PTA)	0.17	0.35	0.49	0.62	–	0.58	0.99	0.59	0.56	–
Anticoagulants										
Warfarin (vs NOACs)	0.75	0.23	3.23	<0.05	2.11 (1.34–3.31)	1.96	0.94	2.08	<0.05	7.10 (1.12–45.15)
None (vs NOACs)	0.69	0.26	2.68	<0.05	1.98 (1.20–3.28)	2.68	0.93	2.87	<0.01	14.51 (2.33–90.24)
Cirrhosis										
No (vs Yes)	−0.56	0.18	−3.05	<0.05	0.57 (0.40–0.82)	−1.87	0.71	−2.63	<0.01	0.15 (0.04–0.62)
Long	−0.08	0.08	−1.01	0.31	–	−0.92	0.36	−2.54	<0.05	0.40 (0.20–0.81)
NEU	0.2	0.08	2.67	<0.05	1.22 (1.06–1.42)	0.22	0.24	0.92	0.36	–
ALB	−0.16	0.1	−1.68	0.09	–	0.54	0.32	1.71	0.09	–
GLU	0.06	0.1	0.63	0.53	–	−0.49	0.33	−1.5	0.13	–
TBIL	0.07	0.07	1.08	0.28	–	−0.1	0.19	−0.56	0.58	–
ApoA	−0.09	0.12	−0.7	0.48	–	−1	0.36	−2.79	<0.01	0.37 (0.18–0.74)
LDH	0.09	0.09	1.02	0.31	–	0.18	0.16	1.18	0.24	–
Log (theta)	0.46	0.3	1.52	0.13	–	–	–	–	–	–

ALB = albumin, CI = confidence interval, GLU = glucose, HV = hepatic vein, IVC = inferior vena cava, LDH = lactate dehydrogenase, NEU = neutrophil count, NOACs = new oral anticoagulants, OR = odds ratio, PT = prothrombin time, TBIL = total bilirubin, TIPS = transjugular intrahepatic portosystemic shunt.

## 4. Discussion

In this study, zero values were close to 70% and all tests for goodness of fit reported that the ZINB regression model fitted the data of BCS recurrence better than the other models. In addition to its superior statistical performance, the use of the ZINB model in this study is supported by clinical plausibility. The excess of zero-recurrence cases may reflect 2 underlying patient subgroups: those who achieved long-term remission after effective intervention and consistent anticoagulation, and those whose recurrence may have gone undetected due to mild symptoms or limited follow-up imaging. These real-world phenomena correspond to the dual structure of the ZINB model, which separately estimates the probability of zero recurrence and the frequency of recurrence among at-risk individuals. Therefore, the model not only fits the statistical characteristics of the data but also aligns with clinically meaningful patient trajectories in BCS. Parameters, such as sex, surgery, anticoagulation therapy, cirrhosis, hospital duration, and ApoA status considerably affected the risk of recurrence. Furthermore, age, sex, anticoagulation, cirrhosis, and NEU were considered as risk factors for BCS recurrence. This rare disease predominantly affects middle-aged individuals around the age of 45 years and exhibits a higher rate of multiple relapses than in older patients. Zhang Jing et al^[29]^ reported that this may be because recurrent symptoms are easy to detect in young patients and they show a strong willingness to proactively seek medical attention. BCS affects women of reproductive age; therefore, it may be associated with the use of oral contraceptives, secondary hormonal abnormalities, and other undiscovered genetic factors. However, Moreno et al and Li et al found no sex-based differences in the pathogenesis of BCS.^[13,30]^ Approximately half of the patients were employed as farmers and workers, indicating that the disease is more likely to occur in relatively underdeveloped areas and low-income, heavy manual labor populations. Further studies are warranted to determine the relationship between a higher number of male patients and sex differences in labor intensity.^[31]^ Increased NEU and decreased AOPA are factors that promote relapse. NEUs produce oxygen radicals and inflammatory mediators that infiltrate the ischemic tissue, release tissue factors, or induce other cells to cause tissue factors to promote thrombosis. HDL protects endothelial cells through its anti-inflammatory, antioxidant, and antithrombotic properties. ApoA is the main structural protein in HDL. Therefore, the ApoA is a protective factor in patients with BCS. In this study, the recurrence group showed significantly prolonged PT and APTT, as well as elevated fibrinogen levels. These findings may indicate underlying hepatic dysfunction, subclinical coagulopathy, or compensatory changes in response to recurrent thrombotic events. Increased use of anticoagulants among recurrent patients could also contribute to these laboratory differences. Although these indicators were not included in the final model due to multicollinearity or regularization thresholds, their potential clinical relevance deserves further investigation.

NOACs, such as Rivaroxaban, Apixaban and Dabigatran, might be advantageous over low-molecular-weight heparin or vitamin K antagonists (VKA) because these drugs can be administered orally. They do not have to be monitored by the international normalized ratio and can directly inhibit coagulation factors, such as Xa and IIa, reduce the rate of thromboembolic events in cardiovascular and stroke diseases, cause fewer anticoagulant–anticoagulant interactions, and do not interfere with the model for end-stage liver disease.^[6,32–34]^ A study by the VALDIG^[35]^ consortium in which 14% of the study patients had BCG showed comparable safety and efficacy comparable to that of VKA. Weizhi Li et al^[36]^ reported that compared with aspirin, rivaroxaban can improve stent patency in patients with BCS after percutaneous intravascular intervention and might not increase massive bleeding. According to Du Xiaofei et al,^[37]^ low-dose rivaroxaban is safe for patients with decompensated liver cirrhosis, which causes no significant bleeding or liver injury. However, their high cost and lack of effective antagonists have hindered their widespread application. NOACs are effective and safe for long-term anticoagulation treatment of BCS and warrant further prospective studies.

PTA combined with stent placement has always achieved excellent long-term patency and survival in most Chinese patients with BCS.^[38]^ Nevertheless, the risk of recurrence in the stent group was higher than that in the simple balloon group. This could be due to the following 3 reasons: only 78 patients underwent stent implantation; however, the number of patients in the balloon group was 6 times higher than that in the stent group. An unbalanced sample size may have led to bias the statistical analysis; Patients undergoing stent implantation usually have a greater number of diseased vessels, more serious stenosis, more wide-ranging vessel disease, and longer vessel disease; and The damage to the vascular endothelium due to the balloon is usually transient and reversible. In contrast, metallic materials and design features on the surface of the stent can cause platelet activation, vascular endothelial inflammatory response, and proliferative response, which can increase the possibility of recurrence. Although stent use appeared to be strongly associated with recurrence in our model, the CI for this estimate was wide, suggesting a high degree of statistical imprecision. This imprecision is likely attributable to the relatively small number of patients who underwent stent implantation. Further studies with larger and better-stratified samples are needed to confirm whether stent use is an independent risk factor for recurrence.

To the best of our knowledge, this is the first single-center retrospective study with a large sample size to investigate the application of ZI models for BCS recurrence. However, our study lacks external validation and requires a large sample size and a multi-center, randomized controlled approach to further validate its clinical significance. In the future, more factors should be considered, including stricture length, stent type, and preoperative and postoperative hepatic or portal vein pressure, to provide evidence-based support for screening for risk factors for BCS. In summary, the zero-inflated model is a robust tool for screening factors that affect BCS recurrence, thereby facilitating the implementation of personalized precision intervention treatment.

## Acknowledgments

The consent of all authors has been obtained for this paper.

## Author contributions

**Conceptualization:** Xiangting Liu, Shengli Li.

**Data curation:** Shengli Li, Cuocuo Wang.

**Formal analysis:** Shengli Li, Lei Wang.

**Investigation:** Muyao Zhou, Hui Wang.

**Software:** Muyao Zhou.

**Supervision:** Hui Wang, Yong Zhou.

**Validation:** Cuocuo Wang, Qingqiao Zhang.

**Visualization:** Qingqiao Zhang.

**Writing – original draft:** Shengli Li, Xiangting Liu, Cuocuo Wang.

**Writing – review & editing:** Qingqiao Zhang, Lei Wang.

## Supplementary Material


