# Supplementary material for: Analysis of risk factors for recurrence of Budd–Chiari syndrome: A retrospective study with zero-inflated model
Source: Medicine (Baltimore). 2025 Nov 21;104(47):e45511. doi: 10.1097/MD.0000000000045511 (PMC12643598; doi:10.1097/MD.0000000000045511)
Supplement: Supplementary file 1 [file medi-104-e45511-s001.docx]

Supplementary Table 1. Summary of missing data

| Variable | Missing (%) |
| --- | --- |
| age | 0 |
| sex | 0 |
| occupation | 0 |
| neutrophil count | 0 |
| platelet count | 0 |
| prothrombin time | 0.6 |
| activated partial thromboplastin time | 0.7 |
| fibrinogen | 0.6 |
| aspartate aminotransferase | 0 |
| alanine aminotransferase | 0 |
| albumin | 0 |
| glucose | 13.3 |
| total bilirubin | 0 |
| apolipoprotein A | 36.7 |
| apolipoprotein B | 36.7 |
| lipoprotein(a) | 36.7 |
| high-density lipoprotein | 32 |
| low-density lipoprotein | 32 |
| cystatin C | 1.3 |
| lactate dehydrogenase | 17.1 |
| alpha-fetoprotein | 17.7 |


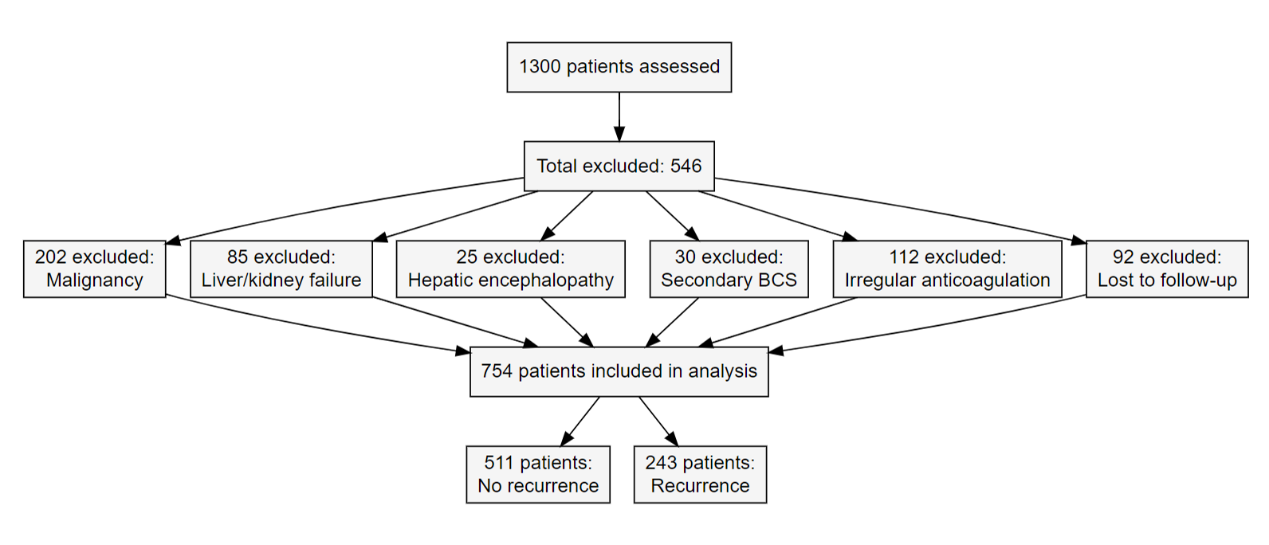


Supplementary figure 1: Patient selection flow diagram.
